# LncRNA NRIR inhibits osteogenesis by promoting macrophage M1 polarization through RSAD2/NF-κB axis in peri-implantitis

**DOI:** 10.3389/fimmu.2025.1650984

**Published:** 2025-10-20

**Authors:** Renshengjie Zhao, Lan Wang, Yang Zhou, Keming Xiao, Qiqi Liu, Ke Yu

**Affiliations:** ^1^ The Affiliated Stomatological Hospital, Southwest Medical University, Luzhou, China; ^2^ Oral & Maxillofacial Reconstruction and Regeneration of Luzhou Key Laborator, Luzhou, China; ^3^ Institute of Stomatology, Southwest Medical University, Luzhou, China

**Keywords:** LncRNA *NRIR*, macrophage polarization, peri-implantitis, osteogenic differentiation, NF-κB, *RSAD2*

## Abstract

**Introduction:**

Peri-implantitis is an inflammatory condition affecting the hard and soft tissues surrounding osseointegrated implants, characterized by progressive alveolar bone destruction. The long non-coding RNA Negative Regulator of Interferon Response (lncRNA *NRIR*) is widely recognized as a biomarker for certain autoimmune diseases and participates in their pathogenesis. However, our previous studies revealed significant upregulation of *NRIR* in peri-implantitis, suggesting its potential role in peri-implantitis. In peri-implantitis lesions, there is often a substantial infiltration of M1 macrophages. Thus, this study investigated the regulatory role and underlying mechanisms of *NRIR* in macrophage polarization during peri-implantitis.

**Methods:**

Transcriptome sequencing analysis revealed radical S-adenosyl methionine domain containing 2 (*RSAD2*) as an *NRIR*-interacting mRNA in macrophages. Using siRNA gene knockdown technology, we suppressed *NRIR* and *RSAD2* expression in M1 macrophages derived from THP-1 cells. Subsequently, we employed RT-qPCR, Western blot, flow cytometry, and immunofluorescence staining to assess the levels of inflammatory cytokines and M1 macrophage-associated markers, aiming to elucidate the involvement of *NRIR*/*RSAD2*/NF-κB axis in macrophage polarization. Supernatants from *NRIR*-knockdown macrophages were collected to prepare the culture medium for bone marrow mesenchymal stem cells (BMSCs). The expression of osteogenic-related factors in BMSCs was evaluated through RT-qPCR, Western blot, Alkaline phosphatase (ALP) activity, and alizarin red S (ARS) staining. Furthermore, a rat peri-implantitis model was established, and the degree of peri-implant tissue inflammation and bone loss was assessed using micro-CT scanning and immunohistochemistry after treatment with various macrophage supernatants.

**Results:**

*NRIR* knockdown reduced *RSAD2* expression and suppressed activation of the NF-κB pathway, consequently decreasing inflammatory cytokines and M1 macrophage-associated cytokine expression in THP-1 macrophages. Functionally, *NRIR* knockdown in macrophages promoted osteogenic differentiation of BMSCs. In vivo experiments showed that supernatants derived from *NRIR*-knockdown macrophages resulted in reduced inflammatory infiltration, diminished bone resorption, and increased expression of osteogenesis-related factors.

**Discussion:**

This study demonstrates that *NRIR* functions as a pro-inflammatory modulator in peri-implantitis by activating M1 macrophages through the *RSAD2*/NF-κB axis, providing novel insights into peri-implantitis pathogenesis that may inform future preventive and therapeutic strategies.

## 1 Introduction

Peri-implantitis is a severe biological complication resulting from bacterial biofilm accumulation in peri-implant tissues, causing inflammation of peri-implant mucosa and subsequent progressive bone loss (1). Over the last 30 years, peri-implantitis has emerged as a significant clinical concern in dentistry (2). Research in osteoimmunology indicates that continuous crosstalk between cells of the monocyte/macrophage/osteoclast lineage and the mesenchymal stem cell-osteoblast lineage determines whether a durable prosthesis-implant interface is established or implant loosening occurs (3). Therefore, exploring interactions between macrophages and mesenchymal stem cells can elucidate the pathogenesis of peri-implantitis.

Macrophages undergo polarization in response to environmental stimuli, with M1 macrophages critically involved in the development of bacterial-induced inflammation, while M2 macrophages contribute to inflammation resolution and tissue repair (4, 5). A large population of M1 macrophages accumulates at sites of bone destruction in chronic osteolytic conditions such as arthritis and periodontitis (6). These macrophages produce substantial amounts of pro-inflammatory cytokines (TNF-α, IL-1, IL-12, IFN-γ), chemokines, and matrix metalloproteinases, inducing osteoclastogenesis, tissue erosion, and progressive bone destruction (7–10). Studies have shown a significant correlation between increased M1 macrophage expression and deeper periodontal probing depths (11). Notably, samples from peri-implantitis lesions exhibit markedly increased M1 macrophage populations (12). This phenomenon likely contributes significantly to the destructive inflammatory response and severe peri-implant osteolysis characteristic of advanced peri-implantitis stages.

In recent years, long non-coding RNAs (lncRNAs) have attracted considerable scientific interest due to their abundance and potential regulatory roles in cellular, molecular, and pathophysiological processes. LncRNAs interact with DNA, RNA, or proteins, thereby regulating transcriptional, post-transcriptional, and translational outcomes (13, 14). Studies on lncRNA functions have suggested their potential as diagnostic/prognostic biomarkers and therapeutic targets in inflammatory diseases, including peri-implantitis and periodontitis (15, 16). LncRNA Negative Regulator of Interferon Response (*NRIR*), an interferon-stimulated gene, is widely considered to play an essential role in the pathogenesis of autoimmune skin diseases, such as systemic sclerosis and systemic lupus erythematosus (17–19). However, few studies have reported the relationship between *NRIR*, macrophage polarization, and peri-implantitis. Further elucidation of these underlying mechanisms will enhance our understanding of peri-implantitis pathogenesis and facilitate the development of preventive and therapeutic approaches.

Our previous studies demonstrated significant upregulation of *NRIR* in peri-implantitis soft tissues (20). The current study further investigates the role of *NRIR* in peri-implantitis-associated macrophage polarization. Through RNA sequencing, we preliminarily identified *RSAD2* as a potential target for *NRIR*. Subsequent *in vitro* experiments revealed that *NRIR* regulates *RSAD2* expression, which in turn influences NF-κB activation and M1 macrophage polarization. Furthermore, supernatants derived from *NRIR*-knockdown macrophages upregulated osteogenic factor expression in bone marrow mesenchymal stem cells (BMSCs) and alleviated inflammation and bone loss in a rat peri-implantitis model. Collectively, this study provides novel insights into peri-implantitis pathogenesis and may inform future strategies for prevention and treatment.

## 2 Materials and methods

### 2.1 Cell culture and stimulation

The THP-1 human monocytic cell line was purchased from Biospecies (Guangdong, China). THP-1 cells were cultured in Roswell Park Memorial Institute 1640 (RPMI 1640, Procell, Wuhan, China) medium supplemented with 10% fetal bovine serum (FBS, PAN, Aidenbach, Germany) and 1% penicillin-streptomycin (Beyotime, Shanghai, China). To induce differentiation into M1 macrophages, THP-1 cells were stimulated with 200 ng/ml phorbol 12-myristate 13-acetate (PMA, Sigma, Shanghai, China) for 24 h to form adherent M0 macrophages, followed by treatment with 100 ng/ml LPS and 20 ng/ml IFN-γ for 48 h.

Human mesenchymal stem cells were obtained from Oricell (Guangzhou, China) and cultured in Minimum Essential Medium α (α-MEM, Procell, Wuhan, China) supplemented with 10% FBS and 1% penicillin-streptomycin.

All cell cultures were maintained in Esco CelMate carbon dioxide (CO_2_) incubators (Esco, Singapore) at 37 °C under 5% CO_2_ conditions to simulate physiological environments.

### 2.2 Cell transfection

Small interfering RNAs (siRNAs) specifically targeting *NRIR* and *RSAD2* were purchased from OBIO (Shanghai, China), with non-targeted siRNA used as negative control (NC). The siRNA and NC sequences are provided in **Supplementary Table S1**. Overexpression plasmids containing full-length RSAD2 and control plasmids were also acquired from OBIO (Shanghai, China); plasmid details are shown in **Supplementary Figure S1**.

For siRNA transfection, 5 × 10^5^ THP-1 cells were seeded into six-well plates and cultured for 24 h, followed by incubation in Opti-MEMI Reduced Serum Medium (31985-070, Gibco, California, USA) for an additional 12 h. Subsequently, 100 pmol of siRNA was transfected into each well using 7.0 μl CALNPTM RNAi reagent A and 2.0 μl CALNPTM RNAi reagent B (D-Nano, Beijing, China).

For plasmid transfection, 5 × 10^5^ THP-1 cells were seeded into six-well plates for 24 h. Upon reaching approximately 80% confluency, 3 μg of plasmid DNA was transfected per well with 4.8 μl Lipofect5000 reagent and 200 μl Trans buffer (BIOG, Changzhou, China).

### 2.3 RNA sequencing

Total RNA was isolated from M1 macrophages using TRIzol reagent (Sangon, Shanghai, China) following the manufacturer’s instructions. RNA quality assessment, library preparation, sequencing, quality control, mapping reads to the reference genome, and differential gene expression analyses were performed by Novogene Co., Ltd (Beijing, China). For functional annotation, Gene Ontology (GO, http://www.geneontology.org/) and Kyoto Encyclopedia of Genes and Genomes (KEGG, http://www.genome.jp/kegg/) analyses were conducted to elucidate gene functions and relevant pathways associated with differentially expressed genes (DEGs). Gene Set Enrichment Analysis (GSEA) was further performed to identify key KEGG signaling pathways. R software (version 4.40) was utilized to visually represent data through principal component analysis (PCA), volcano plots, heatmaps, and pathway enrichment plots. The RNA-seq data were deposited in the NCBI Sequence Read Archive (SRA) (https://www.ncbi.nlm.nih.gov/sra) (BioProject ID: PRJNA1281584; BioSample accession numbers: SAMN49569226, SAMN49569227, SAMN49569228, SAMN49569229, SAMN49569230, and SAMN49569231).

### 2.4 Quantitative real-time polymerase chain reaction

Total RNA was extracted from THP-1 macrophages and BMSCs using the SteadyPure Rapid RNA Extraction Kit (Accurate Biology, Hunan, China). RNA was reverse-transcribed into complementary DNA (cDNA) using the Evo M-MLV RT Mix Kit (Accurate Biology, Hunan, China). Subsequently, RT-qPCR was performed using SYBR^®^ Green Premix Pro Taq HS RT-qPCR Kit (Accurate biology, Hunan, China), following the manufacturer’s instructions. Relative gene expression levels were calculated using the 2^-ΔΔCt^ method as described by Livak et al. (21). Primers synthesized by Sangon Biotech (Shanghai, China) are listed in **Supplementary Table S2**.

### 2.5 Western blotting

Total proteins were extracted from THP-1 cells and BMSCs using a total protein extraction kit (Keygen Biotech, Jiangsu, China). Protein concentrations were quantified using a BCA assay Kit (Beyotime, Beijing, China). After centrifugation (12,000 × g, 4°C, 10 min), protein lysates were separated by 10% or 12.5% SDS-PAGE and transferred onto polyvinylidene fluoride (PVDF) membranes. Membranes were blocked with 5% skimmed milk in Tris-buffered saline containing Tween-20 (TBST) at room temperature for 2 h, then incubated overnight (4°C) with primary antibodies against RSAD2 (Proteintech, 28089-1-AP, 1:1000), CD86 (Huabio, ET1606-50, 1:1000), iNOS (Huabio, HA722031, 1:1000), phospho-p65 (CST, 3033 T, 1:1000), p65 (Proteintech, 10745–1-AP, 1:1000), phospho-IκB (Huabio, HA722770, 1:1000), IκB (Huabio, ET1603-6, 1:1000), Osteopontin (OPN, Proteintech, 80912-4-RR, 1:4000), osteocalcin (OCN, Bioss, bs-4917R, 1:1000), runt-related transcription factor-2 (RUNX2, Huabio, ET1612-47, 1:5000), and GAPDH (Affinity, AF7021, 1:1000). Subsequently, membranes were washed three times in TBST (10 min each) and incubated with HRP-conjugated secondary antibodies (Goat anti-Rabbit IgG, Proteintech, SA00001-2, 1:4000) at room temperature for 2 h. Protein bands were visualized using enhanced chemiluminescence reagent (Affinity, West Virginia, USA) and an iBright CL1000 imaging system (Thermo, MA, USA). Semi-quantitative analysis of protein expression was performed using ImageJ software (v1.8.0, NIH, MD, USA).

### 2.6 Flow cytometry

Following successful macrophage polarization, THP-1 cells were harvested and filtered to prepare single-cell suspensions. Fc receptor-mediated nonspecific binding was blocked using human Fc receptor blocking solution (TruStain FcX™, BioLegend, 422301). Cells were washed three times, stained on ice with PE-conjugated anti-CD86 antibody (Biolegend, 374205) in staining buffer containing 1% FBS, and analyzed using a FACSMelody flow cytometer (BD). Data analysis was performed using FlowJo software (Tree Star).

### 2.7 Immunofluorescence staining

THP-1 cells were fixed in 4% paraformaldehyde (PFA) for 30 min, permeabilized with 0.5% Triton X-100 for 30 min at room temperature (20°C), and blocked with 5% goat serum in phosphate-buffered saline (PBS) for 1.5 h (37°C). Subsequently, cells were incubated overnight (4°C) with primary antibodies against CD86 (Huabio, ET1606-50, 1:500) and iNOS (Huabio, HA722031, 1:100). After three washes with PBS, cells were incubated with fluorescent secondary antibodies for 1 h (37°C, in darkness). Cell nuclei were counterstained with 4′,6-diamidino-2-phenylindole (DAPI, Solarbio, Beijing, China). Stained cells were visualized using laser scanning confocal microscopy (BC43 SR, OXFORD, UK).

### 2.8 Macrophage supernatant preparation

M0 macrophages were transfected and subsequently polarized into M1 macrophages using LPS/IFN-γ for 48 h. Cells were then washed three times with PBS and cultured in serum-free medium for an additional 24 h. SNs were collected, centrifuged (300 × g, 10 min) to remove cellular debris, and filtered (0.22 μm filter). A portion of SNs was combined 1:1 with osteogenic induction medium (Oricell, Guangzhou, China) for BMSC co-culture experiments, and the remainder was used directly for *in vivo* experiments.

### 2.9 Alkaline phosphatase activity and alizarin red S staining

BMSCs underwent osteogenic induction upon reaching 80% confluency. After 14 days, ALP activity was assessed using an ALP staining Kit (Beyotime, Beijing, China). Calcium mineralization was evaluated after 21 days by Alizarin Red S (ARS) staining (Oricell, Guangzhou, China). Chromogenic reactions were visualized using a stereomicroscope (SZN71, SOPTOP, China).

### 2.10 *In vivo* experiment

Thirty male Sprague-Dawley (SD) rats (8 weeks old) were obtained from the Animal Center of Southwest Medical University. All surgical procedures were approved by the Animal Ethics Committee of Southwest Medical University (SWMU20210414) and followed the ARRIVE (Animal Research: Reporting of *In Vivo* Experiments) guidelines.

Detailed surgical procedures were described in our previous studies (22). Briefly, unilateral maxillary first molars of SD rats were extracted under general anesthesia induced by 4% isoflurane inhalation. After a 4-week healing period, rats were anesthetized similarly, and local anesthesia (articaine with 1:100,000 epinephrine; Primacaine, France) was administered at the surgical site. Gingival incisions were made in the maxillary first molar region, and implant sockets were prepared using a reamer (1.6 mm diameter). Customized Ti-6AL-4V screw-type implants (2mm diameter, 3mm thread length, 1.5mm smooth neck) (22) were then inserted. Four weeks were allowed for osseointegration. Seven rats were excluded after four weeks due to death (n = 3) or implant loosening (n = 4). The remaining rats were randomly allocated into four groups using a random number table: (a) control group (n = 5); (b) LPS group (n = 6); (c) si-NC-SN group (n = 6); and (d) si-*NRIR*-SN group (n = 6).

Different treatments were applied to each group. In the LPS group, LPS derived from *P. gingivalis* (1 mg/mL, 10 µL per injection) was injected into the gingival sulcus around implants every three days for two weeks to establish peri-implantitis (23). PBS replaced LPS injections in the control group. Considering rats lack the *NRIR* gene, direct knockdown via vectors (such as nanoparticle- or adenovirus-mediated siRNA transfer) was infeasible. Referring to prior studies (Tahmasebi et al., Ma et al.) (24–26), we chose macrophage-secreted supernatants (SN) as media to investigate *NRIR*’s role in animal models. Rats in the si-NC-SN group received SN from M1 macrophages transfected with non-targeting siRNA, while those in the si-*NRIR*-SN group received SN from *NRIR* siRNA-transfected M1 macrophages. SN (200 µL) was injected into both buccal and lingual gingiva around implants every 3 days for 2 weeks. Rats were sacrificed four weeks post-injection, and tissues were harvested for analysis.

### 2.11 Micro-computed tomography

Maxillae were scanned using micro-CT (Inveon PET CT, Siemens, Germany) with the following parameters: spot size (50 µm), tube voltage (80 kVp), tube current (500 µA), and total rotation (360°). Three-dimensional (3D) reconstructions were analyzed using Mimics (version 21.0) software. Peri-implant bone loss was assessed by measuring the distance from the most coronal marginal bone position to the apical implant head at distal, mesial, buccal, and palatal locations. Bone mineral density (BMD), trabecular thickness (Tb.Th), bone volume fraction (BV/TV), trabecular separation (Tb.Sp), and trabecular number (Tb.N) were quantified according to the micro-CT analysis guidelines outlined by Huang et al. (27).

### 2.12 Immunohistochemical analysis

Maxillary alveolar bone specimens were fixed in 4% paraformaldehyde and decalcified for 6 weeks 10% ethylene diamine tetraacetic acid (EDTA, Solarbio, Beijing, China). Subsequently, specimens were sectioned into 4 µm slices (proximal-distal direction), blocked with 5% BSA for 1h, and incubated with primary antibodies overnight (4°C) according to manufacturer protocols. Slices were then incubated with DAB chromogenic solution, counterstained with hematoxylin (3 min), and visualized under an Olympus BX51 microscope (Tokyo, Japan). Semi-quantitative analysis was conducted using ImageJ software (v1.8.0, NIH, MD, USA). Staining intensity was quantified by calculating average optical density (AOD) as the ratio of integrated optical density (IntDen) of positively stained regions to the area (AOD = IntDen/Area).

### 2.13 Statistical analysis

Statistical significance was assessed using two-tailed Student’s t-test or one-way ANOVA. Where ANOVA showed significant differences, Student-Newman-Keuls q (SNK-q) *post-hoc* tests were performed to identify statistically significant pairwise differences. Variables not meeting normal distribution criteria were analyzed using the Kruskal–Wallis H test. Statistical analyses were performed using SPSS software (version 27.0, SPSS Inc., Chicago, USA). Quantitative data are presented as mean ± standard deviation (SD). Differences were considered statistically significant at P < 0.05.

## 3 Results

### 3.1 LncRNA *NRIR* participates in M1 macrophage polarization in peri-implantitis

In our previous study, high-throughput transcriptome sequencing identified lncRNA *NRIR* in gingival tissues from peri-implantitis patients. *NRIR* may critically contribute to peri-implant inflammation (20). Additionally, seven genes (*RSAD2, CMPK2, IFIT1, IFIT3, ISG15, BST2, and HLA-C*) were predicted as potential *NRIR* targets (20).

**Figure 1:**
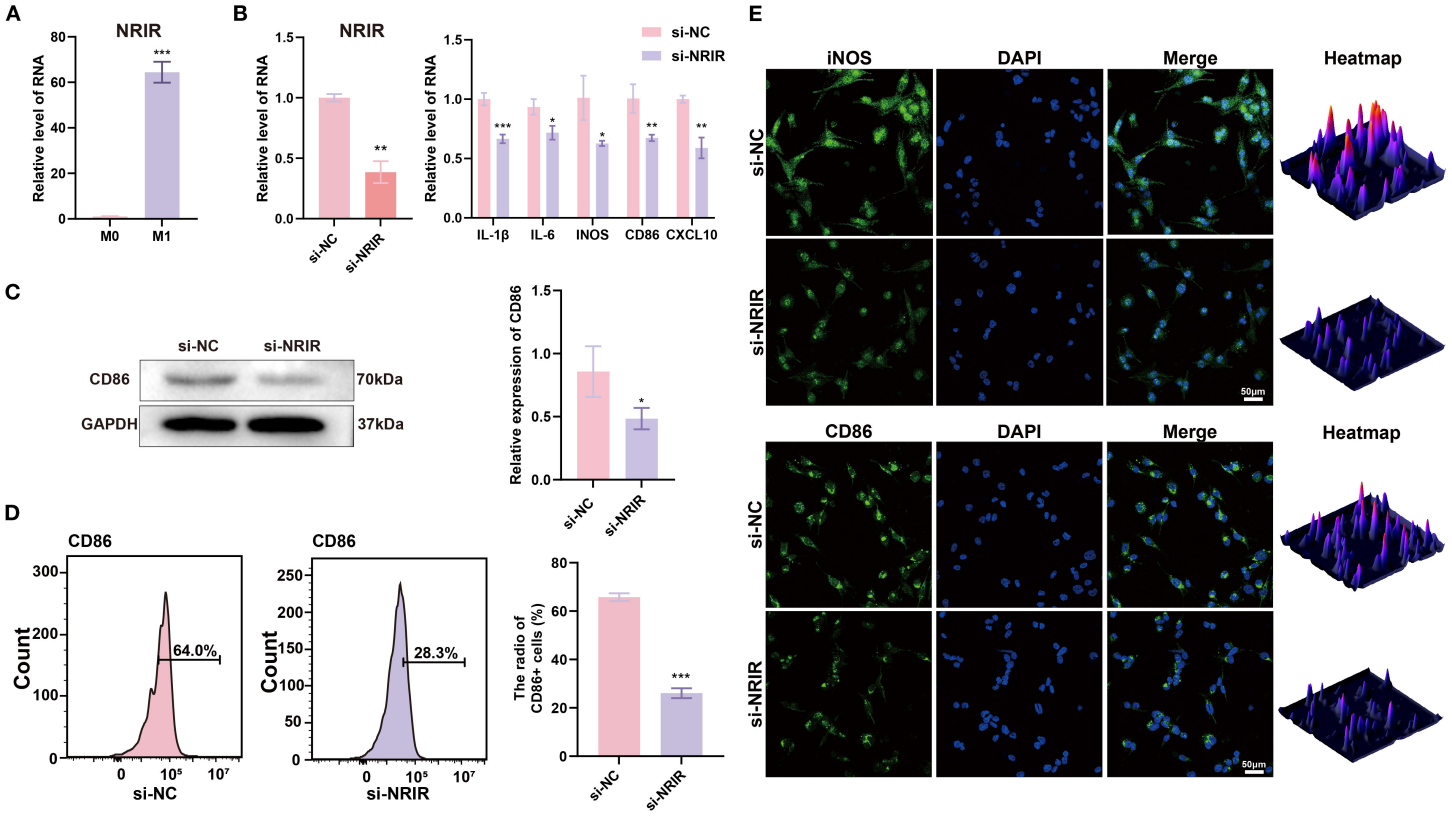
LncRNA *NRIR* involvement in M1 macrophage polarization. **(A)** *NRIR* expression levels in M0 and M1 macrophages. **(B)** RT-qPCR analysis of *NRIR* and M1 polarization-related genes (*IL-1β, IL-6, iNOS, CD86, CXCL10*) after *NRIR* knockdown. **(C)** Western blot analysis of CD86 protein expression after *NRIR* knockdown. **(D)** Flow cytometry evaluation of CD86-positive macrophages following *NRIR* knockdown. **(E)** IF staining of CD86 and iNOS after *NRIR* knockdown. Data represent means ± SD, n = 3; *P < 0.05, **P < 0.01, ***P < 0.001; ns, not significant.

Since macrophages are pivotal immune cells in peri-implantitis, we hypothesized that *NRIR* might regulate macrophage polarization. To test this hypothesis, we analyzed M1 macrophages and observed that *NRIR* was significantly upregulated (**Figure 1A**). Next, we used specific siRNA to silence *NRIR* and evaluated its influence on M1 macrophage activation. RT-qPCR results indicated significant downregulation of M1-associated genes (*IL-1β, IL-6, iNOS, CD86, and CXCL10*) following *NRIR* knockdown (**Figure 1B**). Western blotting demonstrated reduced CD86 protein expression in *NRIR*-knockdown macrophages (**Figure 1C**). Flow cytometry analysis revealed that *NRIR* knockdown significantly decreased M1 polarization, as indicated by CD86 (**Figure 1D**). Additionally, IF assays confirmed that *NRIR* silencing markedly inhibited expression of M1 macrophage polarization markers (iNOS, CD86) (**Figure 1E**).

### 3.2 *RSAD2* is a potential target of *NRIR* regulating macrophage polarization

**Figure 2:**
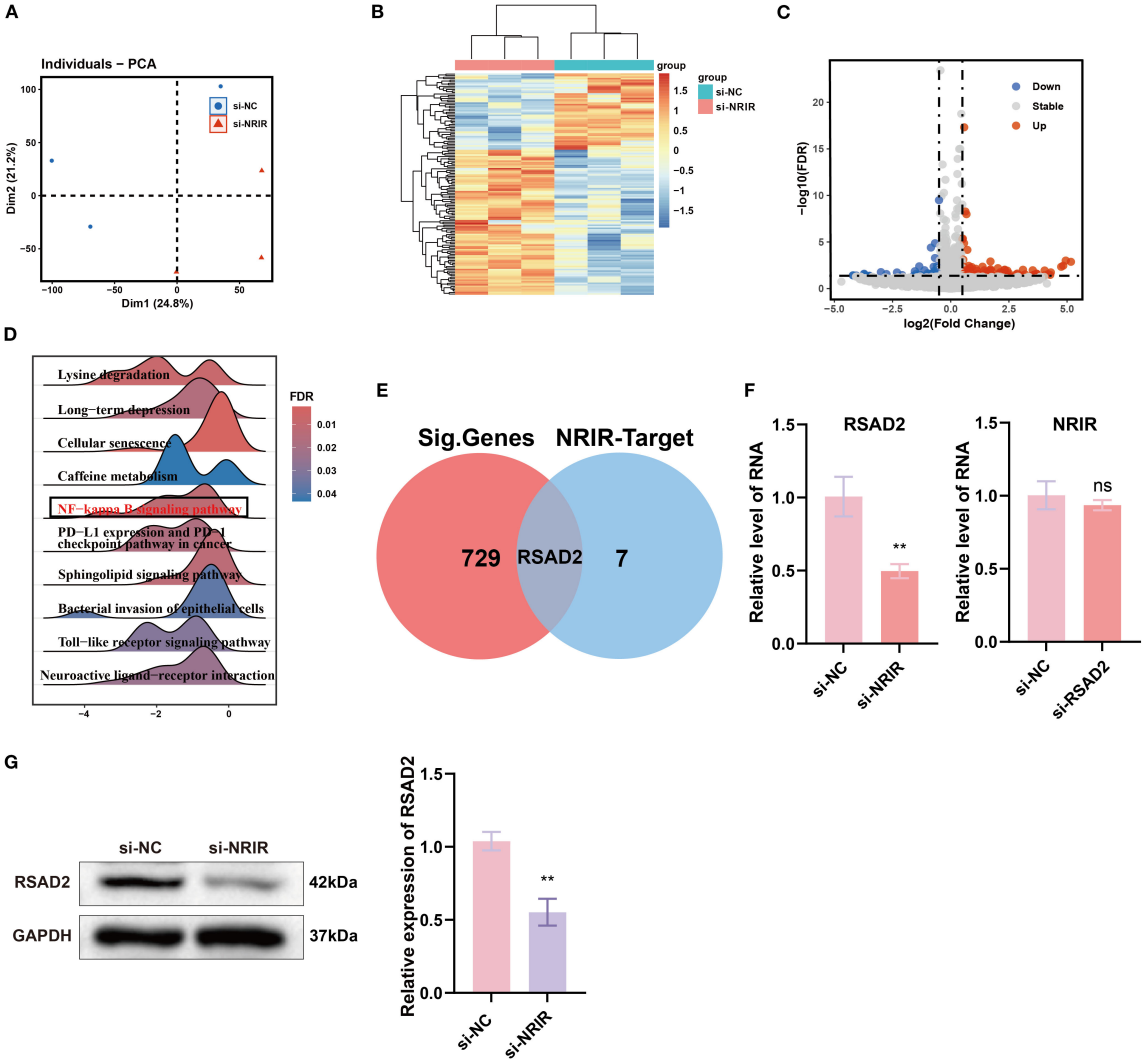
Transcriptomic analysis of macrophage polarization after *NRIR* knockdown. **(A)** PCA plot of transcriptomic profiles. **(B)** Heatmap depicting DEGs. **(C)** Volcano plot illustrating significantly upregulated (red), downregulated (blue), and unchanged (gray) genes. **(D)** GSEA. **(E)** Venn diagram comparing DEGs and predicted *NRIR* targets genes. **(F)** RT-qPCR analysis of *NRIR* and RSAD2 expression. **(G)** Western blot analysis of RSAD2 protein after *NRIR* knockdown. Data represent means ± SD, n = 3; **P < 0.01; ns, not significant.

To further explore the mechanisms underlying *NRIR*-mediated macrophage activation, we performed transcriptome microarray and bioinformatics analyses on M1 macrophages after *NRIR* knockdown. PCA and heatmap analyses demonstrated distinct transcriptomic profiles between control and *NRIR*-knockdown groups, indicating significant gene expression alterations (**Figures 2A, B**). Differential expression analysis identified 729 DEGs (357 downregulated, 372 upregulated), visualized by a volcano plot (**Figure 2C**). GSEA enrichment indicated general downregulation of the NF-κB signaling pathway in *NRIR*-knockdown macrophages (**Figure 2D**). Additionally, intersection analysis between DEGs and predicted *NRIR* targets identified *RSAD2* as the only overlapping gene (**Figure 2E**). Preliminary characterization demonstrated that RSAD2 was downregulated following *NRIR* knockdown at both mRNA and protein levels, whereas *RSAD2* knockdown did not affect *NRIR* expression (**Figures 2F, G**). Thus, we strongly suspected *RSAD2* as a potential target of *NRIR* in macrophage polarization regulation.

### 3.3 *RSAD2* regulates M1 macrophage polarization

**Figure 3:**
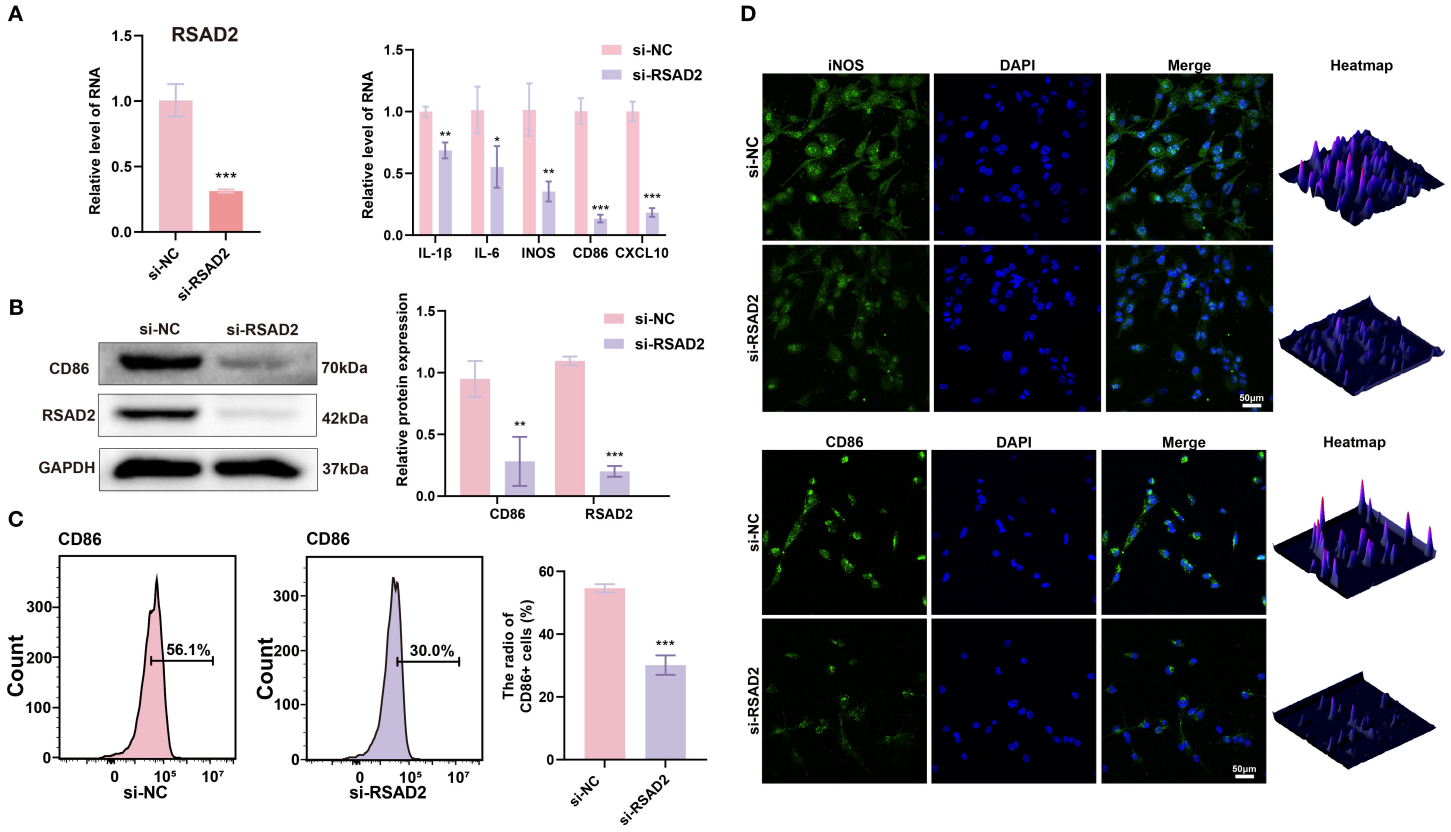
*RSAD2* regulates macrophage polarization. **(A)** RT-qPCR analysis of *RSAD2*, and M1 polarization-related genes (*IL-1β, IL-6, iNOS, CD86, CXCL10*) after *RASD2* knockdown. **(B)** Western blot analysis of RSAD2 and CD86 protein levels after *RSAD2* knockdown. **(C)** Flow cytometry analysis of CD86-positive cells after *RSAD2* knockdown. **(D)** IF staining of CD86 and iNOS after *RSAD2* knockdown. Graphs represent means ± SD, n = 3; *P < 0.05, **P < 0.01, ***P < 0.001; ns, not significant.

To investigate the role of *RSAD2* in M1 macrophages, *RSAD2*-knockdown macrophages were evaluated. At the genetic level, M1-associated genes (*IL-1β, IL-6, iNOS, CD86, and CXCL10*) were significantly downregulated (**Figure 3A**). At the protein level, *RSAD2*-knockdown macrophages showed reduced expression of RSAD2 and CD86 (**Figure 3B**). Flow cytometry results demonstrated that *RSAD2* knockdown markedly reduced M1 polarization (**Figure 3C**). Moreover, IF confirmed that *RSAD2* silencing effectively suppressed the expression of M1 macrophage polarization markers (iNOS, CD86) (**Figure 3D**).

### 3.4 LncRNA *NRIR* promotes M1 macrophage activation by enhancing RSAD2 gene expression

**Figure 4:**
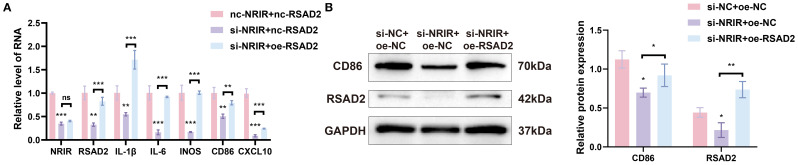
*NRIR* regulates macrophage polarization by enhancing *RSAD2*. **(A)** RT-qPCR analysis of *NRIR*, *RSAD2*, and M1 polarization-related genes after co-transfection. **(B)** Western blot analysis of CD86 and RSAD2 proteins after co-transfection. Graphs represent means ± SD, n = 3; *P < 0.05, **P < 0.01, ***P < 0.001; ns, not significant.

To confirm whether *NRIR* exerts its effects by regulating *RSAD2*, a rescue assay was conducted. Plasmids targeting *RSAD2* were transfected into THP-1 macrophages with confirmed *NRIR* knockdown to restore *RSAD2* expression. Results indicated that *RSAD2* overexpression reversed the reduced gene and protein expression associated with M1 macrophages caused by *NRIR* knockdown (**Figures 4A, B**).

### 3.5 Knockdown of lncRNA *NRIR* inhibits NF-κB signaling by downregulating *RSAD2* during macrophages polarization

**Figure 5:**
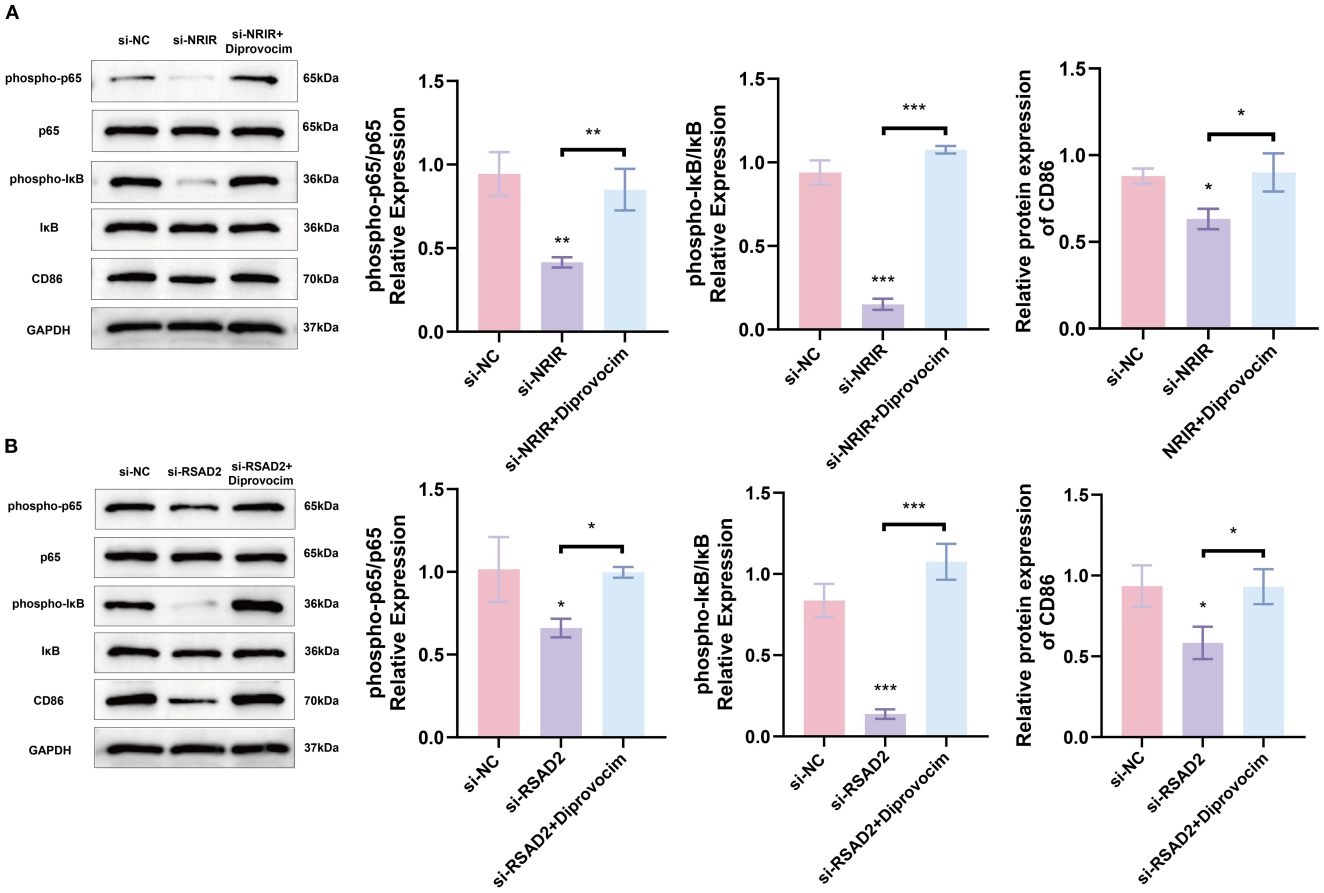
*NRIR* activates NF-KB signaling by regulating *RSAD2*, promoting M1 macrophage activation **(A)** Western blot analysis showing phospho-p65, p65, phospho-IκB, IκB, and CD86 protein levels after *NRIR* knockdown. **(B)** Western blot analysis of the same proteins after RSAD2 knockdown. Data represent means ± SD, n = 3; *P < 0.05, **P < 0.01, ***P < 0.001, ns: not significant.

We subsequently investigated signaling pathways regulated by *NRIR* during macrophage polarization. GSEA indicated that *NRIR* knockdown inhibited the NF-κB signaling pathway (**Figure 2D**). The NF-κB pathway is essential for M1 macrophage activation, particularly in regulating inflammatory gene expression (28). Western blot analysis assessed phosphorylated forms of key factors, IκB and p65. Results showed that *NRIR* knockdown reduced phosphorylated IκB, phosphorylated p65, and CD86 levels in M1 macrophages. However, this reduction was reversed by treating cells with Diprovocim, a potent TLR1/TLR2 agonist that activates NF-κB signaling downstream (**Figure 5A**).

To confirm whether *NRIR* regulates NF-κB signaling via *RSAD2*, we evaluated *RSAD2*-knockdown macrophages. Protein levels of phosphorylated IκB, phosphorylated p65, and CD86 decreased in *RSAD2*-knockdown macrophages during M1 activation. These findings indicate that *NRIR* depletion suppresses NF-kB signaling by downregulating *RSAD2*, further attenuating M1 macrophage polarization (**Figure 5B**).

### 3.6 Macrophage with *NRIR* knockdown promote osteogenic differentiation of BMSCs *in vitro*

**Figure 6:**
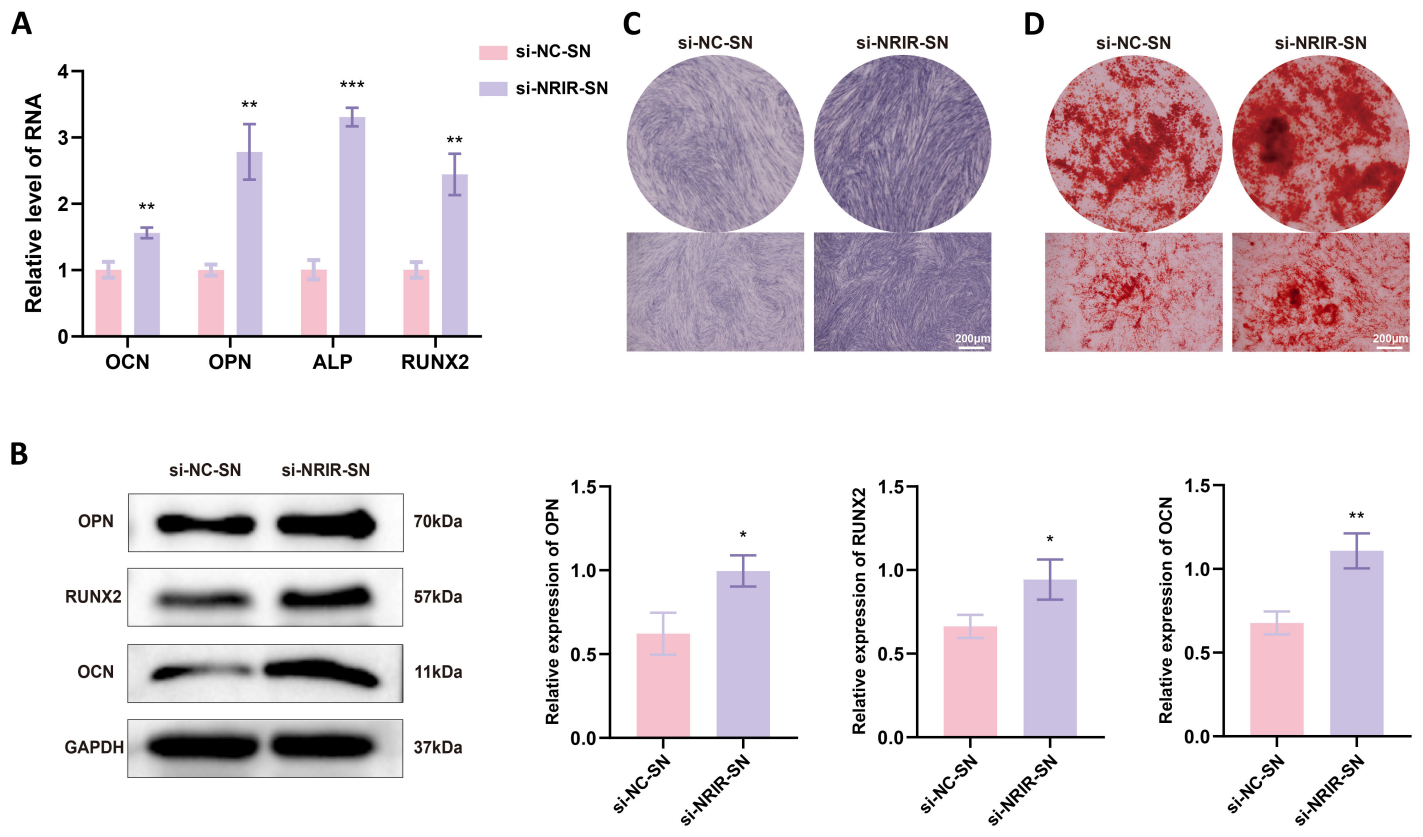
*NRIR* knockdown in macrophages enhances osteogenic differentiation in BMSCs. **(A)** RT-qPCR analysis of *OCN*, *OPN*, *ALP*, and *RUNX2* mRNA levels. **(B)** Western blot analysis of OCN, OPN, and RUNX2 protein levels. **(C)** ALP staining after 14 days of osteogenic induction. **(D)** ARS staining after 21 days of osteogenic induction. Data represent means ± SD, n = 3; *P < 0.05, **P < 0.01, ***P < 0.001.

Chemokines and cytokines secreted by macrophages regulate MSC migration and differentiation for bone regeneration (29–31). To evaluate effects of factors secreted by *NRIR*-knockdown macrophages on BMSC osteogenic differentiation, conditioned medium from *NRIR*-knockdown M1 macrophages was used to culture BMSCs. RT-qPCR and Western blotting showed that *NRIR*-knockdown macrophage supernatants significantly increased expression of osteogenic differentiation markers (OCN, OPN, RUNX2) in BMSCs compared to control groups (**Figures 6A, B**). ALP staining demonstrated enhanced ALP expression in BMSCs cultured with *NRIR*-knockdown macrophage supernatants (**Figure 6C**). ARS staining showed that BMSCs cultured with *NRIR*-knockdown macrophage supernatants formed more mineralized nodules (**Figure 6D**).

### 3.7 Supernatants from *NRIR*-knockdown M1 macrophages reduce inflammation and bone loss in a rat peri-implantitis model

**Figure 7:**
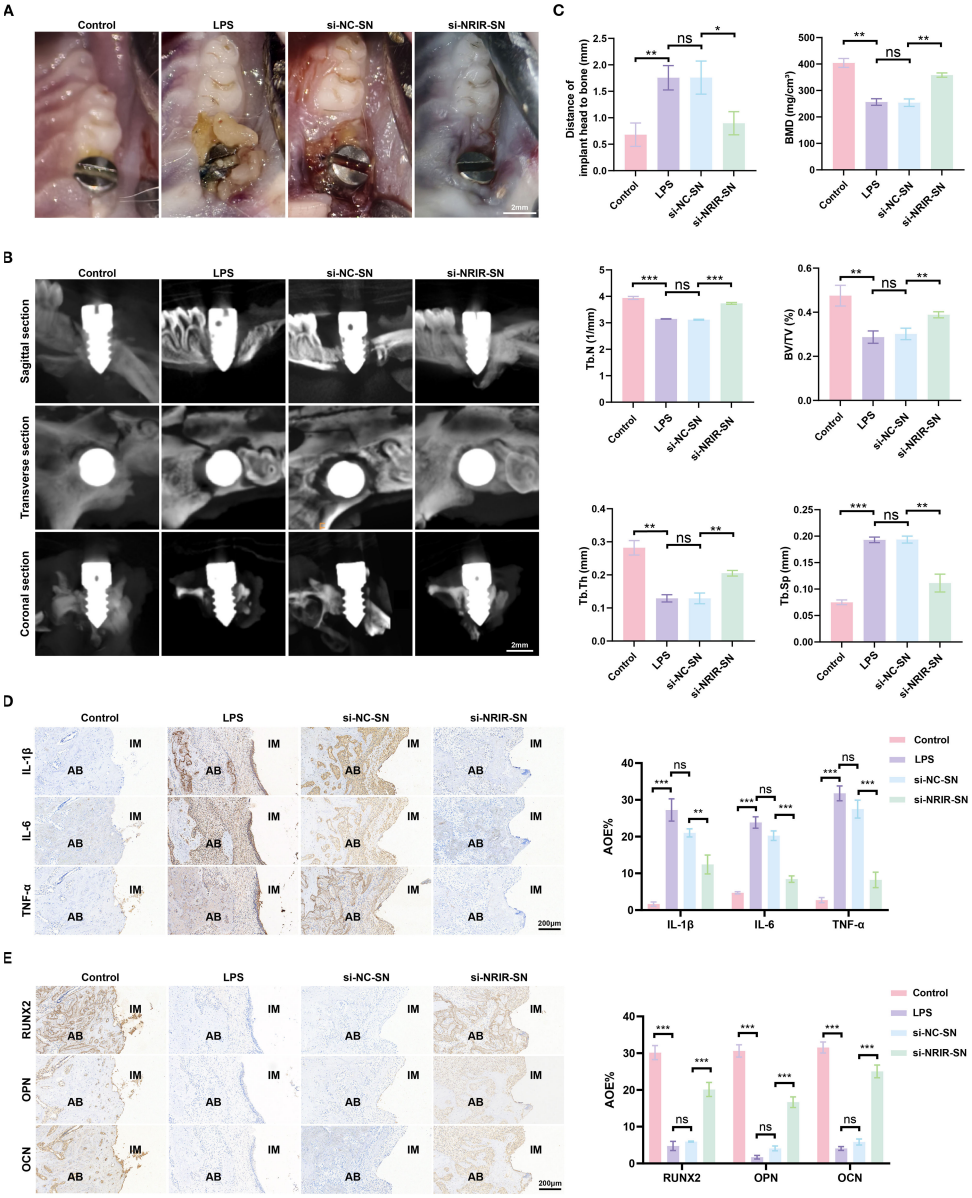
Intraoral images of rats and immunohistochemical analysis of peri-implant tissues. **(A)** Intraoral images from peri-implantitis model rats. **(B)** Maxillary Micro-CT images of each group. **(C)** Bone loss and bone morphometric parameters (BMD, Tb.N, BV/TV, Tb.Th, Tb.Sp) quantified in each group. **(D)** Representative IHC images and semi-quantitative analyses of IL-1β, IL-6, and TNF-α in peri-implant tissues. **(E)** Representative IHC images and semi-quantitative analyses of RUNX2, OPN, and OCN in peri-implant tissues. AB, alveolar bone; IM, implant; AOD, average optical density. Data represent means ± SD, n = 5; *P < 0.05, **P < 0.01, ***P < 0.001, ns, not significant.

Next, we explored the role of *NRIR* in peri-implantitis using a rat model. Clinical observations showed that supernatants from macrophages transfected with si-NC produced clinical manifestations similar to LPS-induced peri-implantitis. However, inflammation was significantly lower in rats treated with supernatants from macrophages transfected with si-*NRIR* (**Figure 7A**). Micro-CT revealed that bone loss in the si-NC-SN group and LPS group was comparable, whereas bone resorption was significantly lower in the si-*NRIR* group (**Figure 7B**). Bone morphometric analysis demonstrated significantly increased BMD, BV/TV, Tb.N, and Tb.Th and decreased Tb.Sp in the si-*NRIR*-SN group compared to the si-NC-SN group (**Figure 7C**). Additionally, histological analyses showed similar inflammatory infiltration and bone resorption in the LPS and si-NC-SN groups, whereas the si-*NRIR*-SN group had reduced inflammatory cytokine expression (IL-1β, IL-6, TNF-α) and elevated osteogenic marker expression (RUNX2, OPN, OCN) (**Figures 7D, E**). Together, *NRIR* knockdown modifies supernatant composition, reducing peri-implant inflammation and bone loss.

## 4 Discussion

**Figure 8:**
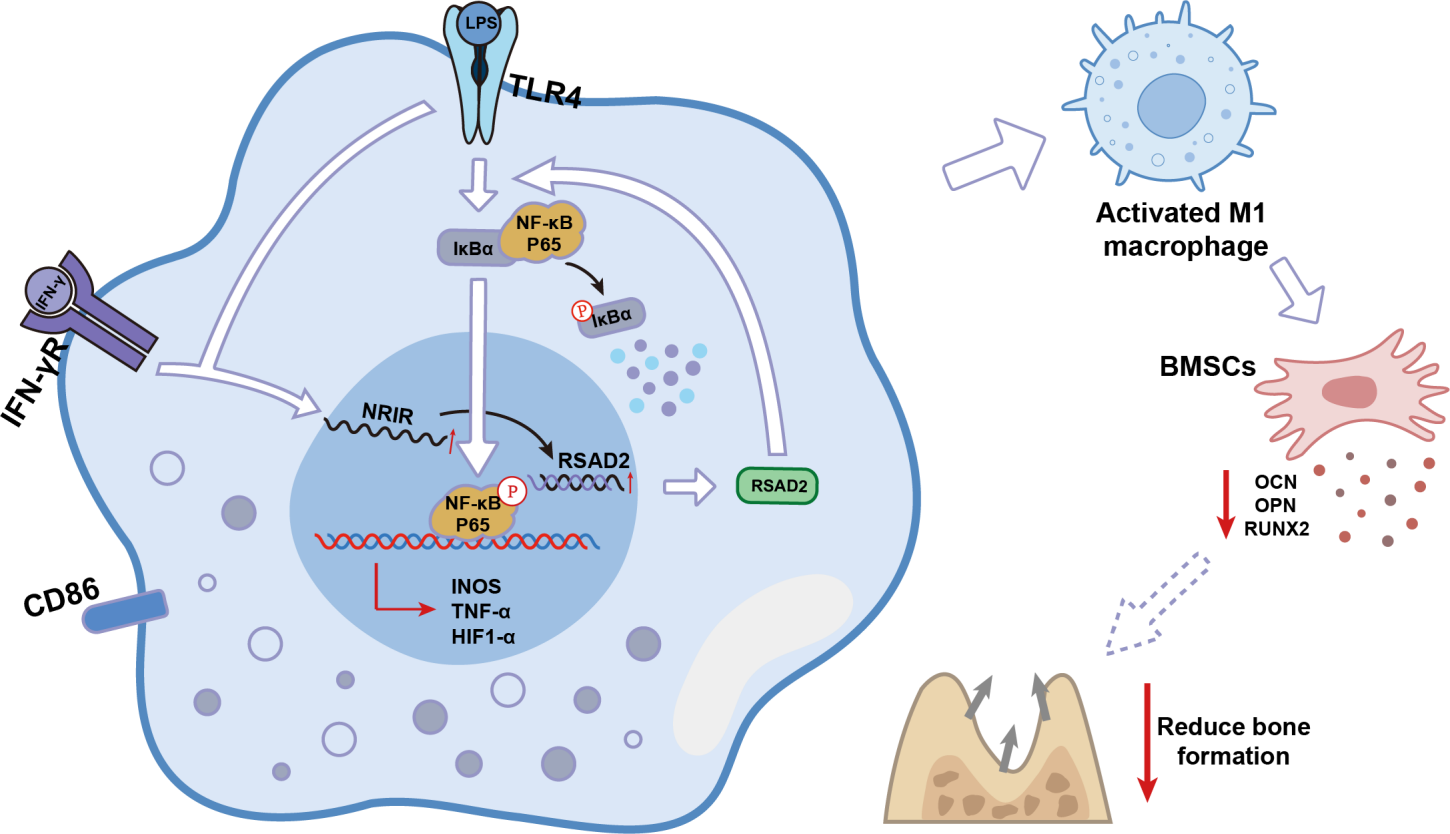
Schematic model of lncRNA *NRIR* functions in M1 macrophage activation.

Over the past few decades, lncRNAs were considered transcriptional “junk” because they do not encode proteins (32). However, recent studies have demonstrated that lncRNAs play critical roles in the activation and function of differentially polarized macrophages in cancer, inflammation, and cardiovascular diseases (19, 33, 34). The present study identified lncRNA-*NRIR*, which regulated *RSAD2* and was highly expressed during M1 macrophage activation. Using loss-of-function approaches, *NRIR* was determined to be a positive regulator of M1 macrophage activation in peri-implantitis. *NRIR* promotes NF-κB activation through *RSAD2*, reduces osteogenesis-related factors in BMSCs, and promotes inflammation-induced bone resorption in peri-implantitis (**Figure 8**). These findings suggest a novel regulatory pathway for M1 macrophage activation, providing new insights into peri-implantitis pathogenesis.

*NRIR* is located on chromosome 2p25.2 and is closely associated with the type I IFN pathway (35). Previous studies have identified *NRIR* as a potential biomarker for systemic lupus erythematosus (SLE), where it may contribute to disease progression (17–19). However, its roles and molecular mechanisms in other diseases, especially macrophage-related conditions, remain poorly understood. A recent report indicated *NRIR* is strongly induced in macrophages during Mtb infection (36). In our study, *NRIR* was similarly induced in LPS/IFN-γ-stimulated M1 macrophages.

*RSAD2* is an interferon-stimulated gene (ISG) encoding the protein viperin, known for antiviral activity (37). Emerging studies emphasize the critical roles of RSAD2/viperin in immunomodulation and mitochondrial metabolism (38–41). Although *RSAD2* expression was significantly increased in polarized human THP-1 and mouse RAW264.7 macrophage models, its precise role in M1 macrophage activation remains unclear (42). Our study revealed that RSAD2/viperin participates in M1 macrophage activation. Moreover, based on previous high-throughput sequencing data and the findings of Cao et al., we verified *NRIR* regulates *RSAD2*, influencing macrophage polarization (20, 43).

As a pivotal inflammatory regulator, nuclear factor kappa-B (NF-κB) promotes the transcription of pro-inflammatory cytokine genes (44). Previous studies confirmed that NF-κB signaling regulates macrophage polarization (45–47). Transcriptome analysis from *NRIR*-knockdown M1 macrophages demonstrated significant down-regulation of NF-κB signaling. Subsequent experiments provided strong evidence that *NRIR* activates NF-κB signaling through *RSAD2*.

The immune and skeletal systems contribute to peri-implantitis by exchanging cytokines, transcription factors, and signaling receptors (48, 49). Macrophages, key immune cells, specifically regulate bone homeostasis. Conversely, BMSCs, major osteoblast progenitors, differentiate into osteoblasts under appropriate conditions, enhancing osteogenesis (50, 51). Pro-inflammatory M1 macrophages secrete various cytokines (TNF-α, IL-1, IL-12, IFN-γ), chemokines, and matrix metalloproteinases, promoting osteoclastogenesis, tissue erosion, and progressive bone destruction (7–10, 52). Based on this principle, supernatants derived from M1 macrophages were utilized to induce peri-implantitis. The results showed that peri-implantitis induced by si-NC macrophage supernatant resulted in a degree of bone loss equivalent to that induced by LPS. Nevertheless, low TNF-α concentrations (20 ng/mL) promote favorable osteogenic outcomes (53). In our experiments, supernatants obtained from *NRIR*-knockdown M1 macrophages enhanced the expression of osteogenic differentiation markers in BMSCs and alleviated inflammation and bone loss around implants *in vivo*. This beneficial effect may be attributed to alterations in cytokine concentrations that promote the differentiation of BMSCs into osteoblasts.

In clinical settings, how to effectively treat patients by targeting *NRIR* is an important consideration. In recent years, gene knockdown has been achieved through RNA interference technology induced by siRNA, opening new avenues for innovative treatments for various diseases (54). Although siRNA-based therapy holds significant promise, its clinical application requires addressing limitations related to targeted delivery, off-target effects, and immunogenicity (55). Several strategies are currently employed to overcome these challenges. Studies have shown that viral vectors, lipid nanoparticles, chemical modifications, or tri-GalNAc conjugates can precisely deliver oligonucleotides to target sites (56, 57). Replacing 2’-O-Me at specific nucleotide sites in the seed region effectively inhibits off-target activity of siRNA (58, 59). Designing siRNA molecules with specific structural modifications can reduce immune activation (60–64). Thus, siRNA targeting *NRIR* represents a promising treatment strategy for peri-implantitis.

However, this study has several limitations. First, the THP-1 cell line used does not fully reflect the behavior of primary macrophages in peri-implantitis. Second, although the rat peri-implantitis model is ideal, it cannot completely replicate human peri-implantitis pathology. Given these limitations and shortcomings, future research should focus on primary human macrophages and further explore these findings through non-human primate models.

In summary, this study elucidates the *NRIR*/*RSAD2*/NF-κB pathway regulating M1 macrophage activation, suggesting potential therapeutic targets for peri-implantitis prevention and treatment. Our findings provide clinically relevant theoretical insights into macrophage polarization regulation associated with peri-implantitis pathogenesis.

## 5 Conclusion

*NRIR* acts as a pro-inflammatory regulator in peri-implantitis. It activates the NF-κB signaling pathway by up-regulating *RSAD2*, promoting M1 macrophage activation, and consequently inhibits osteogenic differentiation in BMSCs. These findings provide novel insights into the pathogenesis of peri-implantitis.

## Data Availability

The data presented in the study are deposited in the NCBI Sequence Read Archive (SRA) repository (https://www.ncbi.nlm.nih.gov/sra), accession number SAMN49569226, SAMN49569227, SAMN49569228, SAMN49569229, SAMN49569230, and SAMN49569231, here: https://www.ncbi.nlm.nih.gov/sra/?term=PRJNA1281584.
