# Supplementary material for: LncRNA NRIR inhibits osteogenesis by promoting macrophage M1 polarization through RSAD2/NF-κB axis in peri-implantitis
Source: Front Immunol. 2025 Oct 20;16:1650984. doi: 10.3389/fimmu.2025.1650984 (PMC12580130; doi:10.3389/fimmu.2025.1650984)
Supplement: Supplementary file 1 [file DataSheet1.pdf]

frontiers

The International Society for the Advancement of Cytometry (ISAC) has highlighted the importance of including comprehensive methodological information to ensure data reproducibility and reliability. In line with this, Frontiers in Immunology now requires authors to submit a checklist for manuscripts that involve flow or mass cytometry. This checklist helps standardize the reporting process, improving the quality and transparency of published data. By doing so, we support scientific progress, making it easier for other researchers to replicate and validate experiments.

This form should be submitted with any manuscripts using flow or mass cytometry.

Sample/specimen/material description

☐ Total blood

☐ PBMCs

☐ Organ digests

Other THP-1 cell line

Did the samples suffer any treatment before or after incubation with the antibodies?

☑ Drug 200ng/ml PMA for 24h, 100ng/ml LPS and 20ng/ml IFN-γ for 48 h.

☐ Cell permeabilization ______

☐ Dye______

☐ Propidium iodine

☐ Not applicable

Other ______

Instrument and antibodies

Name of the Cytometer FACSMelody flow cytometer (BD)

Antibodies and targets	Fluorochrome/ Metal	Catalog number/Company
e.g. anti-CD4	FITC	Cat. XXX/ XXX ltd.
anti-CD86	PE	Cat.374205/Biolegend

human Fc receptor blocking solution		Cat.422301/BioLegend

Data analyses

1. Name of the software FlowJo_v10.9.0
2. Reference gating strategy in the manuscript or supplementary material

Gating strategy in (eg Figure X) We took the sample with only Fc receptor blocking solution added as blank tube. Subsequently, we used the blank tube to determine the negative cell population. Finally, we delineated the negative and positive boundaries on the basis of negative cell populations. (Figure 1D, 3C).
