# Supplementary material for: LncRNA NRIR inhibits osteogenesis by promoting macrophage M1 polarization through RSAD2/NF-κB axis in peri-implantitis
Source: Front Immunol. 2025 Oct 20;16:1650984. doi: 10.3389/fimmu.2025.1650984 (PMC12580130; doi:10.3389/fimmu.2025.1650984)
Supplement: Supplementary file 2 [file DataSheet2.docx]

**
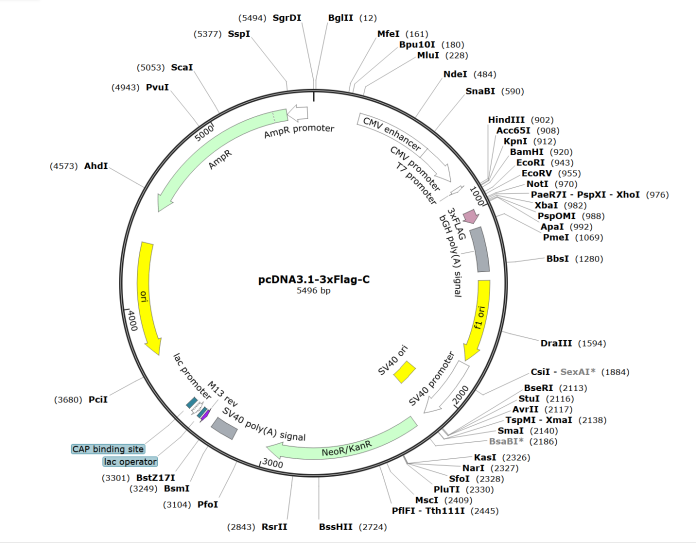

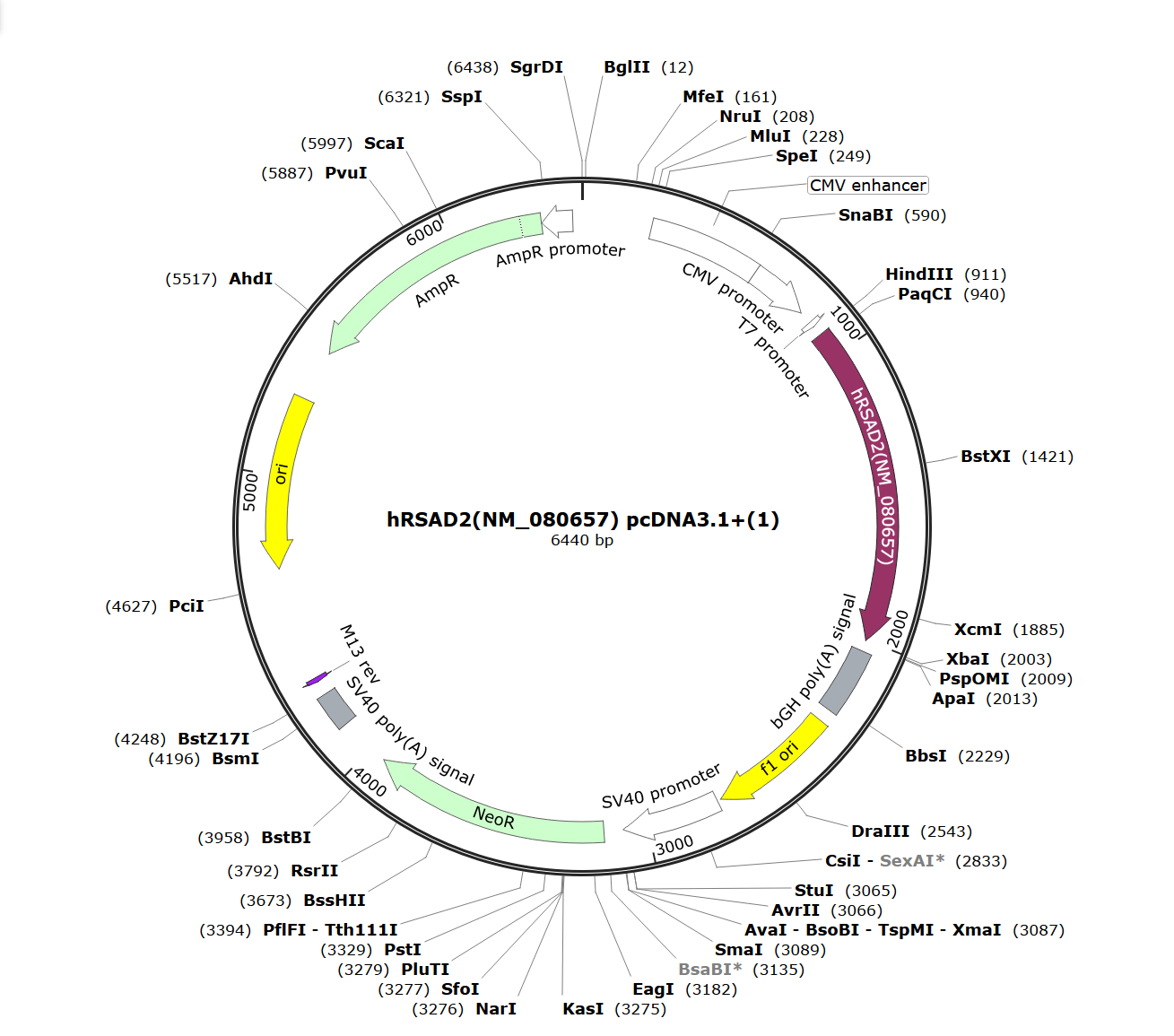
**

Negative control plasmid

plasmid

RSAD2 plasmid

plasmid

**Figure. S1. Plasmid Profile**

**Table. S1. The sequences of siRNAs and negative controls.**

|  | **sense(5' to 3')** | **antisense(5' to 3')** |
| --- | --- | --- |
| NC | UUCUCCGAACGUGUCACGUTT | ACGUGACACGUUCGGAGAATT |
| NRIR | GAUACUAAAGUCAUUUCUACCTT | GGUAGAAAUGACUUUAGUAUCTT |
| RSAD2 | GGUGUAGGGAUUAUAGAGUTT | ACUCUAUAAUCCCUACACCTT |

**Table. S2. The primer sequences for RT-qPCR.**

|  | **Forward Primer(5' to 3')** | **Reverse Primer(5' to 3')** |
| --- | --- | --- |
| IL-1β | CCACAGACCTTCCAGGAGAATG | GTGCAGTTCAGTGATCGTACAGG |
| IL-6 | AGACAGCCACTCACCTCTTCAG | TTCTGCCAGTGCCTCTTTGCTG |
| CD86 | CTGCTCATCTATACACGGTTACC | GGAAACGTCGTACAGTTCTGTG |
| INOS | TCTTGGTCAAAGCTGTGCTC | CATTGCCAAACGTACTGGTC |
| CXCL10 | GAACCTCCAGTCTCAGCACC | GAGAGGTACTCCTTGAATGCCA |
| NRIR | CCACCCCCACGAAGAAATTATATATC | GTTAGAGGTGTCTGCTGCAATAATC |
| RSAD2 | AAAGCTGAGGAGGTGGTGTAGGG | CTTTCCAGCGGACAGGGTTTAGTG |
| OCN | GTGCAGAGTCCAGCAAAGGT | TCAGCCAACTCGTCACAGTC |
| OPN | CATGAGAATTGCAGTGATTTGCT | CTTGGAAGGGTCTGTGGGG |
| RUNX2 | GGAGTGGACGAGGCAAGAGTTT | AGCTTCTGTCTGTGCCTTCTGG |
| ALP | CCCAAAGGCTTCTTCTTG | CTGGTAGTTGTTGTGAGCAT |
| GAPDH | ATGCCTCCTGCACCACCAACT | ATGGCATGGACTGTGGTCATGAGT |
